# SGLT2 Inhibitors: Statins or ACE-Inhibitors of the 21st Century?

**DOI:** 10.3390/jcm12072695

**Published:** 2023-04-04

**Authors:** Michele Correale, Lucia Tricarico, Massimo Iacoviello, Natale Daniele Brunetti

**Affiliations:** 1 Cardiothoracic Department, University Hospital Policlinico Riuniti, 71100 Foggia, Italy; 2 Department of Medical and Surgical Sciences, University of Foggia, 71100 Foggia, Italy

Current guidelines propose therapeutic algorithms based on left ventricular ejection fraction values and clinical presentations; however, these guidelines do not specify which of the four pillar drugs to start first. Some authors suggest starting with SGLT2i and Betablockers, others with ACE-inhibitors or ARNI, while one of the most recent approaches proposes starting with low-dose combination of all four drugs [1].

Eugene Braunwald, distinguished Hersey Professor of Medicine at Harvard Medical School (HMS), editor of *Harrison’s Principles of Internal Medicine* for 12 editions, and founding editor of *Heart Disease*, is the most frequently cited author in cardiology. He is considered one of the leading scientists in the world in research on cardiovascular diseases. In 2021, he compared sodium glucose cotransporter 2 inhibitors (SGLT2i) to statins [2] (drugs capable of delaying the growth of coronary plaques, stabilizing the plaques, and changing the clinical presentation of myocardial infarctions, influencing the prognosis in ischemic heart disease and in the general population). However, considering the most recent updates on SGLT2i, I would compare SGLT2i to ACE-inhibitors rather than statins, mainly due to nephroprotection and the multitarget action of ACE-inhibitors and SGLT2i.

The nephroprotective effect was demonstrated in subsequent studies showing how the eGFR reduction per year was attenuated by dapagliflozin independently of the presence of HF, suggesting an independent nephroprotective action of SGLT2i [3].

Furthermore, SLT2i has been proposed to improve even erythropoietin secretion, improving anemic status, which is typical of CKD patients [3].

SGLT2i are renoprotective, and the protective effects on the kidney seem to predominate over all the other mechanisms of action of SGLT2i inhibitors.

The mechanism of action of SGLT2i in HF is different from that of other HF therapies that focused on reverse left ventricular remodeling, blocking the neurohormonal activation (such as renin-angiotensin-aldosterone system inhibitors), or enhancing the natriuretic peptide system (such as neprilysin inhibitors) [4]. These other classes of drugs have also been shown to have mild favorable kidney effects. Furthermore, reverse left ventricular remodeling with SGLT2 inhibitors is not very significant, despite evidence of their favorable outcome [5].

Other reflections beyond the nephroprotective effect lead us to suggest SGLT2i as a first-line drug in the treatment of HF: 

SGLT-2i does not need up-titration (most HF drugs need titration in order to provide a larger benefit) [3].

SGLT-2i side effects are very minor, making gliflozins extremely well-tolerated drugs [3].

SGLT2i use is not accompanied by hypotension, bradycardia, or hyperkalemia that often accompany the use of currently recommended treatments for heart failure [6].

The cardiologists may consider dose reduction of thiazide or loop diuretics at the time of SGLT2i initiation to avoid excessive diuresis and volume depletion (SGLT2i may induce osmotic diuresis via glucosuria) [6].

SGLT2i treatment has been demonstrated to improve cardiovascular outcomes in patients with HF over a wide range of LVEF, regardless of diabetic status, and has a strong renoprotective effect [5]. In fact, data from DELIVER and EMPEROR-Preserved TRIAL with SGLT2i in patients with HFpEF encourage the use of SGLT2i for the pharmacological approach to HFpEF [5]. 

Inhibition of renal glucose reabsorption affects blood pressure and improves the hemodynamic profile and the tubule glomerular function [5].

Natriuresis derived by Empagliflozin is not associated with neurohormonal activation, potassium loss, or renal impaired function (i.e., a favorable diuretic profile) [5].

The benefits of SGLT2i may also be due to favorable hemodynamic and metabolic effects on LV [7]. The direct effects of SGLT2i on the endothelial and vascular function may contribute to hemodynamic effects [7,8].

The addition of SGLT2i to OMT in patients with HFrEF resulted in a greater improvement in RV systolic function [9].

Finally, recognition by physicians that the benefits of SGLT-2i use on clinical outcomes outweigh the risks will result in the integration of SGLT-2is into clinical practice and lead to improved patient care and outcomes [10].

## Data Availability

Not applicable.
